# The clinical impact of preoperative biliary drainage on isolated infectious complications (iiC) after pancreatic head resection—a retrospective study

**DOI:** 10.1186/s12893-021-01366-1

**Published:** 2022-02-26

**Authors:** Colin M. Krueger, Sophia Chikhladze, Ulrich Adam, Maciej Patrzyk, Axel Kramer, Hartwig Riediger

**Affiliations:** 1 Department of Surgery, Immanuel Clinic Rüdersdorf, University Clinic of Brandenburg Medical School, Seebad 82/83, Rüdersdorf b, 15562 Berlin, Germany; 2 Department of General and Visceral Surgery, Faculty of Medicine, Medical Center - University of Freiburg, Freiburg im Breisgau, Germany; 3 Department of General Surgery, Vivantes-Humboldt Hospital, 13509 Berlin, Germany; 4 Department of Surgery, Clinic of General, Visceral, Vascular and Thoracic Surgery, University Medicine Greifswald, Greifswald, Germany; 5 Institute of Hygiene and Environmental Medicine, University Medicine Greifswald, Greifswald, Germany

**Keywords:** Pancreatic surgery, Bacteriobilia, Infectious complication, Surgical site infection, Preoperative biliary drainage

## Abstract

**Background:**

The perioperative morbidity after pancreatoduodenectomy (PD) is mostly influenced by intraabdominal complications which are often associated with infections. In patients with preoperative biliary drainage (PBD), the risk for postoperative infections may be even elevated. The aim of this study is to explore if isolated infectious complications without intraabdominal focus (iiC) can be observed after PD and if they are associated to PBD and antibiotic prophylaxis with potential conclusions for their treatment.

**Methods:**

During a 10-year period from 2009 to 2019, all consecutive PD were enrolled prospectively in a database and analyzed retrospectively. Bacteriobilia (BB) and Fungibilia (FB) were examined by intraoperatively acquired smears. A perioperative antibiotic prophylaxis was performed by Ampicillin/Sulbactam. For this study, iiC were defined as postoperative infections like surgical site infection (SSI), pneumonia, unknown origin etc. Statistics were performed by Fisher’s exact test and Mann Whitney *U* test.

**Results:**

A total of 426 PD were performed at the Vivantes Humboldt-hospital. The morbidity was 56% (n = 238). iiC occurred in 93 patients (22%) and accounted for 38% in the subgroup of patients with postoperative complications. They were not significantly related to BB and PBD but to FB. The subgroup of SSI, however, had a significant relationship to BB and FB with a poly microbial profile and an accumulation of *E. faecalis*, *E. faecium*, *Enterobacter*, and *Candida*. BB was significantly more frequent in longer lay of PBD. Resistance to standard PAP and co-existing resistance to broad spectrum antibiotics is frequently found in patients with iiC. The clinical severity of iiC was mostly low and non-invasive therapy was adequate. Their treatment led to a significant prolongation of the hospital stay.

**Conclusions:**

iiC are a frequent problem after PD, but only in SSI a significant association to BB and FB can be found in our data. Therefore, the higher resistance of the bacterial species to routine PAP, does not justify broad spectrum prophylaxis. However, the identification of high-risk patients with BB and PBD (length of lay) is recommended. In case of postoperative infections, an early application of broad-spectrum antibiotics and adaption to microbiological findings from intraoperatively smears may be advantageous.

## Background

During the last decades, mortality rates after pancreatoduodenectomy (PD) decreased significantly due to improved treatment options. In specialized centers, double-digit mortality rates are a thing of the past. Nevertheless, morbidity is still high and surgical complications are the main issue [1–3]. In patients with preoperative biliary drainage (PBD), the risk of postoperative infectious complications may be even elevated [4, 5]. Surgical site infections (SSI) after PD are often found to be in relation to preoperative biliary drainage (PBD) and associated bacteriobilia (BB) [6–8]. We suspect that other postoperative infections like pneumonia or infections of unknown origin may be associated with PBD, too. The incidence of these isolated infectious complications without abdominal/surgical focus (iiC) could be elevated in high-risk patients with PBD and BB.

The aim of this work is to analyze the presence of iiC after PD in relation to preoperative biliary interventions.

## Methods

In this study, all consecutive PD from the decade of 1/2009 to 12/2019 at the Vivantes-Humboldt Hospital in Berlin were investigated.

Preoperative Endoscopic Retrograde Cholangiopancreatography (ERCP) was performed either according to internal standards for a serum bilirubin > 8 mg/dl or for other indications at the decision of the gastroenterologist. For this study, the time interval between ERCP and date of surgery was investigated. No information on date of examination were available for 25 patients with external pretreatment. In some patients, ERCP had occurred earlier without substantive reference to PD. Therefore, a subgroup analysis was performed to define patients with a time interval of 60 days or less between ERCP and PD. No patient underwent percutaneous biliary drainage instead of ERCP.

The standard perioperative antibiotic prophylaxis (PAP) was Ampicillin/Sulbactam (AS) or, in case of penicillin allergy, ciprofloxacin and metronidazole by i.v. infusion and 30 min before surgical skin incision. A second dose was administered when the duration of surgery was > 4 h. Prophylaxis of surgical site infections (SSI) was performed according to the 24 recommendations of the Center for Disease Control (CDC) [9, 10]. In case of postoperative clinical evidence of infection, empirical treatment with Piperacillin/Tazobactam (PT) was regularly performed, which was later adjusted accordingly based on the results of the biliary smear or the clinical situation.

This biliary smear was taken routinely, intraoperatively and aseptically obtained from the bile fluid after transection of the ductus hepatocholedochus (intraBD). For subsequent evaluation, the most common species (*E. faecalis, E. faecium, S. pneumoniae, Klebsiella spp., E. coli, and Enterobacter spp.*) were analyzed in detail. All other species were grouped as rare pathogens. This subdivision into common and rare pathogens based on the results of a previous study [11]. Biliary flora with more than one bacterial species was classified as polymicrobial. Detection and non-detection of bacterial species were classified as BB + or BB-, if not; and fungi (fungibilia, FB) were scored as FB + and FB−. With the detection of at least one resistant species in the biliary flora to AS (AS resistant) or PT (PT resistant) and this individual was counted as resistant in subgroup analysis. For this study, data on the postoperatively administered antibiotic treatment were not analyzed individually.

In the present study, preoperative cholestasis was defined as PC + (> 1.2 mg/dl) or PC− (< 1.2 mg/dl) based on the normal value of serum bilirubin. Preoperative biliary drainage was recorded as PBD + or PBD−.

Postoperative pancreatic fistula (POPF) was classified according to international guidelines [12, 13]. All complications were classified following the Accordion classification [14]. The six-level system differentiates into ward-level measures, and invasive interventions, organ failure and death. All infectious complications were prospectively documented in the patients’ charts study database.

For this study, we reviewed the prospectively documented cases from our database. We defined the occurrence of postoperative clinical infection without concomitant evidence of intra-abdominal focus was defined as an isolated infectious complication (iiC). According to the definitions, no patient with iiC had a clinically relevant type B/C fistula and no patient with iiC had a surgical, abdominal complication. SSI were counted, when clinical signs of infection or reopening of the skin or subcutaneous tissue without relaparotomy (A1 and A2 according to CDC) occurred. Pneumonia was defined from the clinical/radiological data. Infection of unclear etiology existed when there were elevated laboratory infection parameters without clinical or radiological finding of a focus. Clinical relevant subgroups of iiC were developed in a retrospective supplement.

Perioperative and microbiological data were collected in a local database (IBM SPSS Statistics, IBM, version 25). Cross table analysis was applied by utilizing Fisher’s exact test while the Mann–Whitney U test was used for non-parametric analysis.

## Results

During the study period 426 PD were performed. As a standard procedure, pylorus-preserving pancreatoduodenectomy (PPPD) was performed most frequently (n = 358; 84%) and classic Whipple surgery was performed in only a few selected cases (n = 68; 16%). Indications were malignancies (n = 302, 71%), chronic pancreatitis (n = 64, 15%), and other benign diseases (n = 64, 15%), such as cystic neoplasms. The median patient age was 69 years (259 male: 167 female). Morbidity was 66% (n = 238), mortality 3.8% (n = 16). The incidence of POPF was 20% (n = 86), and leakage of the biliodigestive anastomosis occurred in 1% (n = 5).

Twenty-two percent of patients (n = 93) had iiC and patients with iiC accounted for 38% of all patients with complications.

### ERCP

ERCP was performed in 82 patients with a serum bilirubin level above 8 mg/dl and in 130 patients for different reasons. In total, preoperative ERCP with PBD occurred in 50% of patients (n = 212). At time of surgery, PC + was present in 42% of patients (n = 180) with 117 of them having undergone ERCP. BB + was present in 54% of patients (n = 231).

With biliary drainage in place, BB was significantly clustered (PBD + : n = 180 (78%) vs. PBD−: n = 32 (16%); p < 0.01), the bacterial profile was significantly more frequent polymicrobial (poly + (n = 141; 66%) vs. poly- (n = 71; 27%); p < 0.01) and FB was significantly more frequent FB + (n = 31; 91%) vs. FB- (n = 181 (46%); p < 0.01). Detection of FB + was rare in PBD-/PC- (n = 1/153; 0.7%), PBD−/PC + (n = 2/63; 3%), PBD + /PC- (n = 7/93; 8%), but significantly heaped in PBD + /PC + (n = 24; 21%); p < 0.01.

The median interval between ERCP and surgery was 27 d (1–326). Subgroup analysis of patients with PBD duration < 60d (n = 171) showed a high rate of BB already in the first two weeks (0–14 days: 70/88; 80%) but also a significant increase over the course (15–60 days: 76/83; 92%); p = 0.03. In detail, there was a significant increase in *Enterococcus* (36/88 (41%) vs. 52/63 (63%); p < 0.01), *E. faecium* (5/88(6%) vs. 17/83 (21%); p < 0.01), *Escherichia* (11/88 (13%) vs. 24/83 (29%); p = 0.01) and *Enterobacter* (8/88 (9%) vs. 19/83 (23%); p = 0.02).

However, the incidence of iiC was independent from the length of lay of PBD (0–14 days: 19/88 (22%) vs. 15–60 days: 19/83 (23%); p = 0.83).

### iiC

In terms of clinical aspects and according to the study criteria, iiC were divided into infections of unclear etiology (n = 38), SSI (n = 24), and Pneumonias (n = 20) and others (n = 18). PBD itself did not have an association to the occurrence of iiC. BB and FB, however, were found significantly more often in patient with SSI, and FB were more frequently in the biliary flora of patients with fever of unknown origin (Table 1).

**Table 1:** Number and subgroups of iiC (n = 93) after PD (n = 426) with reference to PBD, BB and FB

Type of iiC	All patients 426 n (%)	PBD + /PBD-212/214 n (%)/n (%)	BB + /BB-231/195 n (%)/n (%)	FB + /FB-34/392 n (%)/n (%)
All	93 (21)	48 (22)/45 (21)	54 (23)/39 (17)	**13 (38)/80 (20)**
Unknown origin Wound infection (SSI) Pneumonia Colitis Cholangitis Urinary tract infection Venous Catheter associated infection	38 (9) 24 (6) 20 (5) 8 (2) 7 (2) 3 (1) 1 (0)	20 (9)/18 (8) 15 (7)/9 (4) 8 (4)/12 (6) 3 (1)/5 (2) 5 (2)/2 (1) 2 (1)/1 (0) 0 (0)/1 (0)	23 (10)/15 (11) **18 (8)/6 (3)** 9 (4)/11 (5) 3 (1)/5 (2) 5 (2)/2 (1) 2 (1)/1 (0) 0 (0)/1 (0)	**7 (21)/31 (8)** **6 (18)/18 (5)** 1 (3)/19 (5) 0 (0)/8 (2) 0 (0)/7 (2) 0 (0)/3 (1) 0 (0)/1 (0)

Percentage figures in parentheses refer to the total in line1, bold = p < 0.05

When iiC occurred, the only heaped biliary species were *E. coli* (iiC + : 21/93 (23%) vs. iiC−: 41/333 (12%); p = 0.02) and *Candida spp.* (iiC + : 13/93 (14%) vs. iiC−: 21/333 (6%); p = 0.03).

SSI was found in 24 patients and in 21 of these patients, additional microbiological findings from the wound were available. SSI was significantly heaped in the presence of BB (Enterobactales and grampositive species) and FB. In the biliary flora, the species *E. faecalis, E. faecium, Enterobacter and Candida spp.* were significantly frequent (Table 2). In patients with SSI, microbiological findings from wound smears showed identic bacterial species in 38% of patients (n = 8) and fungi in 10% of patients (n = 2), when compared to microbiological findings of intraoperative smears.

**Table 2:** Bacterial profile of the bile in patients with SSI (n = 24) after PD

	SSI + (n = 24) n (%)	SSI− (n = 402) n (%)	p
Bacteriobilia (n = 231)	18 (75%)	213 (53%)	0.04
PBD (n = 212)	15 (63%)	197 (49%)	0.21
Enterobactales (n = 124)	12 (50%)	112 (28%)	0.02
Escherichia (n = 62)	6 (25%)	56 (14%)	0.14
Enterobacter (n = 36)	5 (21%)	31 (8%)	0.04
Klebsiella (n = 60)	5 (21%)	55 (14%)	0.36
Grampositive (n = 179)	15 (63%)	164 (41%)	0.03
*E. faecalis* (n = 131)	12 (50%)	119 (30%)	0.04
*E. faecium* (n = 37)	6 (25%)	31 (8%)	0.01
*S. pneumoniae* (n = 67)	3 (13%)	64 (16%)	1.00
Rare bacterias (n=267)	14 (58%)	253 (63%)	0.67
Polymicrobial (n=165)	15 (63%)	150 (37%)	0.01
Multiresistant (n=10)	1 (4%)	9 (2%)	0.4
Candida (n=34)	6 (25%)	28 (7%)	<0.01

Patients grouped by Enterobactales (Escherichia, Enterobacter, Klebsiella) and grampositive species (Enterococcus, *E. faecium*, *S. pneumoniae*)

### Antibiotics

In patients with BB (n = 231), the biliary flora was AS-resistant (n = 110) and in almost every second patient out of this group PT-resistant (n = 60), too. Vice versa, from 61 patients with resistance to PT, 60 had a resistance to AS, too. From 170 patients with no resistant species to PT, a resistance to AS was found in 50 individuals. However, routine PAP with AS had a weakness compared to PT in a relevant number of patients. It should be noted, that the bacterial flora of only one out of 61 patients showed a resistance to PT but not to AS (Table 3).

**Table 3:** Analysis of the bacterial flora in patients with BB (n = 231)

	At least one species PT-resistant (n = 61)	No species PT-resistant (n = 170)	
At least one species AS-resistant (n = 110)	60	50	p = 0.01
No species AS-resistant (n = 121)	1	120	

Full coverage of all bacterial species by PT vs. AS, respective finding of at least one species with resistance to one of each antibiotics

In addition to the results from Table 3, a focus was set on patients with different types of iiC. In subgroup analysis of all patients with iiC and resistance to AS, the co-existing resistance to PT was further analyzed. Here, a high portion of PT-resistance was detected. This was the case in all entities of iiC, except pneumonia.

Following to that, in SSI a resistogram of microbiological from wound smears was available in 13 patients. Here, the analyzed species were both PT-resistant and AS-resistant and only in one case sensitive to both substances.

### Treatment

In most cases, therapy of iiC required basic, non-invasive treatment. Patient with iiC were significantly more often classified as Accordion grade 1 and 2 (iiC + n = 74; 79% vs. iiC−; n = 82; 25%; p < 0.01). However, severe courses were also observed.

iiC resulted in a significant prolongation of the hospital stay. The median postoperative hospital length of stay for patients without complications was 15 d (Rage 7–30). For patients with iiC, it was significantly longer 25 d (7–167); (p < 0.01). Patients with SSI could be discharged after a mean of 18 d, with infections of unclear origin after 20 d and patients with pneumonia after 40 d.

## Discussion

PD is associated with a high perioperative morbidity [2, 3]. Surgical complications and associated postoperative infections are most relevant. Infectious complications are often related to the occurrence of intra-abdominal abscesses and surgical complications like anastomosis insufficiency [4, 15, 16]. The risk of postoperative infectious complications may be even higher after PD with PBD and consecutive BB [17, 18]. Consequently, the indication for stent implantation should only be given in the presence of cholangitis or relevant cholestasis (> 8 mg/dl at our clinic). Otherwise, surgery should better be performed promptly [18, 19].

Even if we are strict about the indication for ERCP, our data show that in this current patient population, one out of two patients had PBD prior to surgery. To our mind, in relevant cholestasis, restoration of a regular liver function is required preoperatively. Above a value of 5 mg/dl there may be a relevant restriction of liver function and biliary drainage indicated [20]. However, even with PBD, a relevant number of patients from our study have bilirubin level above normal (PC +) at the day of surgery. With an average interval of 27 d between ERCP and surgery, complete normalization of serum bilirubin is achieved in only less than 50% of patients. In other studies, complete normalization of serum levels has been achieved after four to six weeks [21]. At present, it remains unclear, if patients with moderate cholestasis and PBD are at increased risk for iiC in comparison to patients with normal bilirubin levels. Our own data suggest that a longer lay of PBD is on a higher risk for biliary colonization. For clinical practice, optimal coordination (short interval) of ERCP and date of surgery, considering normalization of liver functions, seems to be important to reduce the risk of BB [22]. If PBD was placed, an adequate liver function should be used as the trigger to find the earliest possible surgical date.

According to our definition, only patients with iiC (without intra-abdominal focus) are included in this study. An analysis of the overall morbidity or surgical complications as anastomotic insufficiencies is not performed here. We hypothesize that patients with iiC represent a special group after PD in relation to PBD. The underlying pathophysiological theory is an occult bactieremia, that may cause postoperative infections.

To generate a high study volume, we decided for a retrospective analysis of the prospectively documented patient related data. We are aware of the basic limitations of a retrospective approach but consider it justified as the idea of analyzing iiC is new. The retrospective design may lead to an underestimation of SSI and a potential discrimination bias in patients with clinical and radiological inapparent intraabdominal postoperative abscess. In order to give more precise statements about the significance of iiC in future analyses, it will be necessary to evaluate the risk profile of patients preoperative. This refers especially to potential infectious risk factors like COPD, antibiotic history, recurrent biliary stent treatments, etc.

In the overall group, approximately one in five patients (93/426; 22%) suffer from iiC and iiC account for more than one-third of all patients with complications (93/238; 38%). An association of iiC and BB respective FB could only be shown in case of SSI.

Depending on the study, SSI are defined clinically both with and without germ detection [16, 23, 24]. Here, we could obtain results from the species in the wound in 21/24 (88%) cases. Like shown in the literature, in case of SSI, species are often the same as in bacteriobilia [7]. Our data demonstrate an association between PBD + associated BB + and the occurrence of SSI. *E. faecalis, E. faecium*, and *Enterobacter* predominate as common species in case of SSI. *Candida spp.* were also found to be accumulated in the biliary flora in SSI. An increased rate of SSI following to PBD must be assumed according to various studies [5, 16, 18, 25, 26]. A significant accumulation of both Enterobactales and grampositive species was found in the biliary flora of patients with SSI. In only one patient with SSI, a multiresistant species was found. Whether additional clinical relevant information can be concluded from the new concept of multi-, extensively or pandrug resistance must be investigated in further studies [27].

However, 9/24 patients had SSI without having PBD and this leads to assumption that other risk factors may exist. Next to the reduction of PBD, other actions have been explored to reduce the risk of SSI.. A reduced rate of SSI has been shown with at least 72 h of perioperative application of a broad-spectrum antibiotic [15]. Another option may be the use of intensified local infection prevention measures in high-risk subgroups. One promising option is the use of sutures impregnated with antimicrobial substances like Triclosan. In evaluating the current published evidence, CDC recommends the suture material with high evidence in abdominal surgery, laparotomy and appendectomy for deep abdominal closure and fascia with absorbable suture material [28]. The preventive effect has been also confirmed for PD in a controlled clinical trial [29]. Further important results have been demonstrated in the BaFo Trial where a beneficial effect on SSI was shown by using circular plastic wound protectors during surgery [30, 31]. In addition to that, negative pressure wound therapy over closed incisions is a novel approach to prevent SSI for laparotomy wounds including after PD at appropriate patient-selection [32]. An incisional negative pressure dressing has been demonstrated to reduce SSI following PD especially for patients with highest infection risk [6].

Preoperative adjustment of perioperative antibiotic prophylaxis (PAP) depending on the presence of PBD is not yet recommended by the German Therapy Guidelines [19]. However, some studies propose this approach because of an increased perioperative morbidity in the presence of a higher rate of resistance in the biliary flora [33]. The data and recommendations regarding extended PAP are currently still inconsistent or, to our mind, in favor of having broad-spectrum antibiotics in reserve in case of an infection. An undirected escalation of perioperative antibiotic prophylaxis also does not seem to be justified on the basis of fundamental microbiological considerations [11]. The data of Tables 3 and 4 support our clinical approach to treat postoperative infections with a broad-spectrum antibiotic like PT, because the biliary flora is typically more often resistant to standard PAP. In our clinical routine we adapt the antibiotic treatment escalation according to the resistogramm.

**Table 4:** Subgroup analysis of iiC: Biliary flora of patients with at least one resistant species to PAP (AS-resistant). Therefrom patients with at least one or no species with resistance to PT

Groups of patients with infectious complications	Patients with bacterial resistance to AS n	Therefrom		
		Patients with at least one PT resistant species n (%)	Patients with no PT resistant species n (%)	p
All iiC (n = 93)	29	19 (66%)	10 (34%)	< 0.01
Unknown origin (n = 38)	13	10 (77%)	3 (23%)	< 0.01
SSI (n = 24)	13	8 (62%)	5 (38%)	< 0.01
pneumonia (n = 20)	2	2 (10%)	0 (0%)	n.a

*n.a.* not applicable due to constant values

## Conclusions

Patients with iiC account for more than one third of all patients with complications after pancreatic head resection. Except from SSI, the suspected association of PBD, BB, and FB to iiC and its subgroups could not be found in our data. SSI are the second largest group of iiC and their treatment causes a significant prolongation of the hospital stay in our data.

Limiting the duration of length of lay of PBD will have a beneficial effect on the of the rate of BB. Based on the shown data, we do not recommend a blanket escalation to a broad-spectrum antibiotic in prophylaxis of SSI. The appliance of local therapies may be further evaluated in high-risk patients. In case of postoperative infections, an early application of broad-spectrum antibiotics and adaption to microbiological findings from intraoperatively smears will be advantageous.

## Data Availability

The datasets used and/or analyzed during the current study are available from the corresponding author on friendly request.

## Abbreviations

iiC: Isolated infections complications
PBD: Preoperative biliary drainage
BB: Bacteriobilia
FB: Fungibilia
